# Comments on Rithidech, K.N.; *et al.* Lack of Genomic Instability in Bone Marrow Cells of SCID Mice Exposed Whole-Body to Low-Dose Radiation. *Int. J. Environ. Res. Public Health* 2013, *10*, 1356–1377

**DOI:** 10.3390/ijerph10072732

**Published:** 2013-07-02

**Authors:** Keith Baverstock

**Affiliations:** Department of Environmental Sciences, University of Eastern Finland, Kuopio Campus, Kuopio, Finland; E-Mail: keith.baverstock@uef.fi

I would like to take issue with Rithidech *et al*., authors of the paper entitled “Lack of genomic instability in mice at low doses” [1] who claim to have shown that their results on the measurement of late occurring chromosome aberrations after irradiation of SCID mice with X-rays show that lower doses (0.05 Gy) do not induce genomic instability. Their earlier work at higher doses (0.1 and 1.0 Gy) on the same strain of mouse indicated that *de novo* chromosome aberrations were detected at 6 months post-irradiation. This was taken, almost certainly correctly, to be an indication of the presence of genomic instability: late appearing chromosome damage, as the authors note, seems to be a reliable indicator of the process. The lack of *de novo* chromosome aberrations at 6 months post-irradiation, however, cannot be taken as evidence of the absence of genomic instability. In drawing their conclusion of a “lack of genomic instability ….” the authors have committed two category errors.

The chromosome aberrations the authors *observed* are: (a) a *material* manifestation and (b) a modification of the *genotype*, whereas, the genomic instability they *inferred* is (a) a *process* and (b) a modification of the *phenotype*. The first error is that by concluding that a lack of late occurring chromosomal aberrations indicates a lack of genomic instability they have conflated a material object (chromosomal aberrations) with a process (genomic instability): the former may well be an indicator of the latter but its absence cannot be assumed to be evidence of the absence of the latter. To take an analogous example let us consider another process, apple ripening, for which in many varieties a red skin is an indicator of the process occurring, but the definitive evidence is increasing concentration of sugars in the fruit. However, many varieties of apple, if shielded from light, ripen (increases in sugar content) without acquiring a red skin. Thus, a red skin is an indicator of ripening but the absence of red skin is not evidence of the absence of ripening.

The trap that authors have fallen into in the second category error is to assume that the modification to the genotype is the cause of the modification to the phenotype. This is, in a sense, understandable because it is part of the prevailing biological dogma, but genomic instability is a problem precisely because it does not comply with that dogma [2,3,4]. Indeed, the phenomenon itself is evidence that the upward (genotype to phenotype) causation is *not* applicable. In the experimental work that uncovered the phenomenon [5] clones derived from single alpha-irradiated cells contained both cells with a normal karyotype and cells with chromosomal aberrations. This can only be interpreted (unless it can be assumed that the damage was reversible) as indicating that the first divisions of the irradiated cell did not exhibit the aberrations and that they occurred *de novo* in the later divisions. In other words the instability phenotype caused the aberrations by downward causation. Consider another earlier example of the phenomenon [6]: f_0_ male mice injected with ^239^Pu and mated with untreated females produced an excess of intrauterine foetal death (IUFD), taken to be due to a dominant lethal (DL) mutation. However, surviving f_1_ males, without further ^239^Pu exposure, and mated with untreated females also produced an excess of IUFD. Clearly, a DL mutation cannot, by definition, skip a generation so this evidence is best interpreted as genomic instability being induced in the f_0_ or f_1_ generations and appearing as IUD in the f_1_ and f_2_ generations. This is a strong indication that genomic instability is not a genetic, but an epigenetic effect [3,4,7]. So, in the paper under discussion [1] the genomically unstable phenotype (which originates either in the irradiated cell or its immediate progeny) and not the radiation directly, caused the chromosomal modifications to its own genotype the authors observed. Downward causation is of course contrary to the prevailing dogma but the case for upward causation is deeply flawed in other ways [8,9] and the very phenomenon of genomic instability forces us to reassess the ground rules of not just radiobiology, but biology [3,10,11] as well.

To observe, rather than infer, the absence of genomic instability it would be necessary to show that the phenotype has not been changed by the radiation exposure, that is, show that the active proteome is unmodified. I suspect that this is not possible with today’s technology so in practical terms lack of modification of the transcriptome is the best available option. However, it cannot be assumed that the proteome mirrors the transcriptome [12] and so even that it is not a definitive test. However, modification of the transcriptome is good evidence of the presence of the genomic instability [13].

The authors may argue that at some points in their paper [1] they qualified their conclusion of a lack of genomic instability with the words “as determined by the absence of increases in the frequencies of late-occurring CAs in BM cells collected at 6 mo after exposure of ……”. However, they fail to explain the basis for this qualification so the reader is not in a position to judge whether their flawed inference is valid or not.

The public health dimension of this paper concerns the question of whether there is a dose threshold for the induction of cancer or not. In fact, the epidemiological evidence from radiation exposed populations leaves little doubt that for cancer the dose response relationship is linear down to about 0.01 Gy and it seems reasonable to assume that LNT applies in practice as well as theoretically for radiation protection purposes.

With these considerations in mind it is my contention that the paper by Rithidech *et al.* [1] is seriously flawed and should be withdrawn.
